# Antiplatelet strategy during ad-hoc percutaneous coronary intervention in elective patients: how aggressive should we be?

**DOI:** 10.1007/s12471-023-01825-9

**Published:** 2023-10-20

**Authors:** Abi Selvarajah, Renicus S. Hermanides

**Affiliations:** Department of Cardiology, Isala Hospital, Zwolle, The Netherlands

Over the last decade, there has been an increase in the prevalence of ad-hoc percutaneous coronary intervention (PCI) among patients with coronary artery disease (CAD) [1]. This upward trend can be attributed to the effectiveness of primary PCI in acute coronary syndrome (ACS) and studies suggesting ad-hoc PCI is safe and effective compared with delayed PCI, by circumventing potential delays associated with staged interventions, which results in reduced radiation exposure, greater cost-effectiveness and fewer access site-related complications. Nevertheless, determining the most appropriate antiplatelet regimen for patients with stable CAD undergoing ad-hoc PCI remains debatable and uncertain.

The current European Society of Cardiology (ESC) Guidelines on revascularisation recommend the administration of aspirin with a loading dose of clopidogrel in patients undergoing elective PCI, once a comprehensive assessment of coronary anatomy has been made and a decision to proceed with PCI has been definitively reached (Class Ia) [2]. Pretreatment with dual antiplatelet therapy is also recommended in staged procedures within the planned treatment regimen. These guidelines do not explicitly mention an optimal antiplatelet strategy for ad-hoc PCI, as there is no compelling evidence for a significant clinical benefit of routine P2Y_12_ inhibitor pretreatment. Instead, they suggest that pretreatment with clopidogrel may be considered in selected patients who are highly likely to undergo PCI (Class IIb, Level C). Moreover, the small but real chance there is a need to proceed to urgent coronary artery bypass grafting due to unfavourable coronary anatomy often leads to reluctance in physicians to pre-treat their patients with clopidogrel. This hesitation stems from the fact that it typically takes 5 days for platelet function to be fully restored after discontinuation of clopidogrel, which may lead to an anticipated increase in peri- and postoperative bleeding risks [3].

Because of these considerations, ad-hoc PCI is frequently avoided when patients have not previously received P2Y_12_ inhibitor therapy. There are instances where ad-hoc PCI is still performed, with P2Y_12_ inhibitor loading occurring immediately following the PCI. To mitigate unwanted delays and bridge the gap of delayed optimal P2Y_12_ inhibition by administering oral P2Y_12_ inhibitors, the utilisation of an intravenous antiplatelet agent emerges as a favourable alternative. Cangrelor, an intravenous P2Y_12_ inhibitor, has been shown to reduce PCI-related ischaemic complications without increasing major bleeding in patients undergoing urgent or elective PCI [4]. These outcomes have not only been observed in controlled clinical trials, but also in real-world clinical practice [5].

Platelet glycoprotein IIb/IIIa inhibitors (GPIs) are an additional alternative. The FABOLUS-FASTER trial has presented evidence suggesting that tirofiban—owing to its greater potency and consistent inhibition of platelet aggregation—may exhibit superior efficacy in mitigating the risk of acute ischaemic complications compared with cangrelor [6]. It is essential to note that the latter study was conducted in the context of ACS. Therefore, the applicability of tirofiban as a viable option in the context of ad-hoc PCI in elective patients requires further investigation and confirmation. The current ESC Guidelines recommend GPIs only to be considered in specific bail-out situations (Class IIa, Level C) [2].

In the current issue of the *Netherlands Heart Journal*, Habibi et al. retrospectively assessed the safety and efficacy of a single bolus of tirofiban followed by a 600-mg loading dose of clopidogrel (immediately post-PCI) in 432 clopidogrel-naïve patients undergoing ad-hoc PCI by comparing them with 972 patients undergoing elective PCI who were pre-treated with clopidogrel only [7]. The primary endpoint of the study were major adverse cardiovascular events (MACE), defined as a composite of death, myocardial infarction, stroke, target-lesion revascularisation and major bleeding (Bleeding Academic Research Consortium type ≥ 3) at 30 days.

The study showed no statistically significant difference in the occurrence of MACE between the 2 groups [7]. However, it is noteworthy that the patients who received clopidogrel pretreatment had a higher incidence of major bleeding events. This discrepancy may be attributed to their older age and greater risk profile, as well as the fact that more transfemoral procedures were performed in the cohort with clopidogrel pretreatment. In conclusion, the authors posit that ad-hoc PCI in patients who have not previously been exposed to clopidogrel and were treated with a single bolus of high-dose tirofiban, followed by a loading dose of clopidogrel immediately after PCI, appears to be both safe and effective.

Habibi and colleagues must be appreciated for addressing the need for an optimised antithrombotic therapy in a substantial cohort of patients in our routine clinical practice. While the authors imply that their findings may pave the way for the potential utilisation of GPIs in the context of ad-hoc PCI in patients with stable CAD, it is imperative to acknowledge several notable methodological limitations inherent to this study. Its retrospective design, the lack of randomisation and the dissimilarity between the 2 groups characterised by distinct treatment strategies (planned versus unplanned PCI) are crucial factors that warrant consideration. Additionally, the absence of essential angiographic lesion characteristics further adds to the limitations of this investigation.

Future research must adopt a prospective and randomised approach to truly ascertain the optimal antiplatelet strategy for patients with stable CAD undergoing ad-hoc PCI. Such studies should encompass 4 potential strategies for evaluation: (1) pre-PCI clopidogrel loading, (2) post-PCI clopidogrel loading, (3) tirofiban bolus administration, or (4) cangrelor bolus administration. Furthermore, to gain a comprehensive understanding of the additive value of high-dose tirofiban, it is essential to conduct studies focusing on the diverse angiographic characteristics of lesions (for instance, recent plaque ruptures in non-calcified lesions) in patients with stable CAD. These investigations will illuminate the nuanced complexity of antiplatelet therapy in ad-hoc PCI and further contribute to evidence-based clinical decision-making.
